# Diagnosis and management of a heterotopic pregnancy and ruptured rudimentary uterine horn

**DOI:** 10.1186/s40738-018-0051-7

**Published:** 2018-09-29

**Authors:** Paula C. Brady, Rose L. Molina, Michael G. Muto, Brenna Stapp, Serene S. Srouji

**Affiliations:** 1 Department of Obstetrics, Gynecology, and Reproductive Biology, Brigham and Women’s Hospital and Harvard Medical School, Boston, MA 02115 USA; 2 Division of Global and Community Health, Department of Obstetrics and Gynecology, Beth Israel Deaconess Medical Center and Harvard Medical School, Boston, MA 02215 USA; 3 Department of Obstetrics and Gynecology, Elliot Hospital, Manchester, NH 03103 USA

**Keywords:** Heterotopic pregnancy, Mullerian anomaly, Unicornuate uterus, Uterine rupture, Three-dimensional ultrasound

## Abstract

**Background:**

Heterotopic pregnancies implanted in a rudimentary uterine horn account for 1 in 2–3 million gestations, and confer significant risk of morbidity due to uterine rupture and hemorrhage.

**Case presentation:**

A 34-year-old nullipara presented with acute pelvic pain at 17 weeks of gestation with dichorionic-diamniotic twins, one in each horn of an anomalous uterus first diagnosed in pregnancy as bicornuate. Three-dimensional ultrasound and MRI revealed myometrial disruption in the left rudimentary uterine horn, and the patient underwent an uncomplicated abdominal hemi-hysterectomy. Fourteen days later, an uncomplicated dilation and curettage was performed for a fetal anomaly in the remaining twin in the right unicornuate uterus.

**Conclusion:**

This case demonstrates the utility of magnetic resonance imaging and three-dimensional ultrasound in the assessment of myometrial integrity in a gravid patient with a heterotopic pregnancy and ruptured rudimentary uterine horn. This case demonstrates the importance of pre-pregnancy diagnosis and management of mullerian anomalies.

## Background

Mullerian anomalies occur in up to 4% of women, but may be asymptomatic and go undetected [1]. Unicornuate uteri account for just 5% of these anomalies, or 1 in 4,000 women [1]. Most unicornuate uteri (83%) are accompanied by rudimentary horns, most of which are non-communicating [2]. In current practice, magnetic resonance imaging (MRI) and three-dimensional (3-D) ultrasound are the optimal noninvasive imaging modalities for diagnosis of mullerian anomalies; nonetheless misdiagnosis of a rudimentary horn is not uncommon [1, 3].

Pregnancy outcomes vary with the type of mullerian anomaly; gestations implanted in rudimentary uterine horns—accounting for 1 in 100,000 to 140,000 pregnancies—are associated with particularly high risk of morbidity [1, 3]. Eighty percent or more of gravid rudimentary uterine horns will rupture (usually before 24 weeks of gestation), resulting in hemorrhage and requiring emergency surgery, with a 0.5% maternal mortality rate [3, 4].

Heterotopic pregnancies involving two uterine cavities—one fetus in the rudimentary horn and one in the accompanying unicornuate uterus—account for just 5% of pregnancies implanted in rudimentary uterine horns [4]. These pregnancies occur at a rate of 1 in 2–3 million pregnancies. This obstetrical complication has been reported rarely in the past 20 years (likely due to the advancement of imaging technologies), and without MRI and 3-D ultrasound imaging [5–7].

## Case presentation

The patient is a 34 year old healthy primigravida with spontaneous dichorionic diamniotic twins and likely bicornuate uterus, with one fetus in each horn, initially diagnosed at 12 weeks gestation by two-dimensional ultrasound. Magnetic resonance imaging at that time reported bicornuate unicollis uterine anatomy, with symmetrical myometrial thickness in both horns. The left cervical canal was noted to communicate with the right lower uterine segment and not directly with the vagina. A thin amniotic band was noted in the superior left uterine horn. Bilateral normal maternal kidneys were noted.

**Fig. 1:**
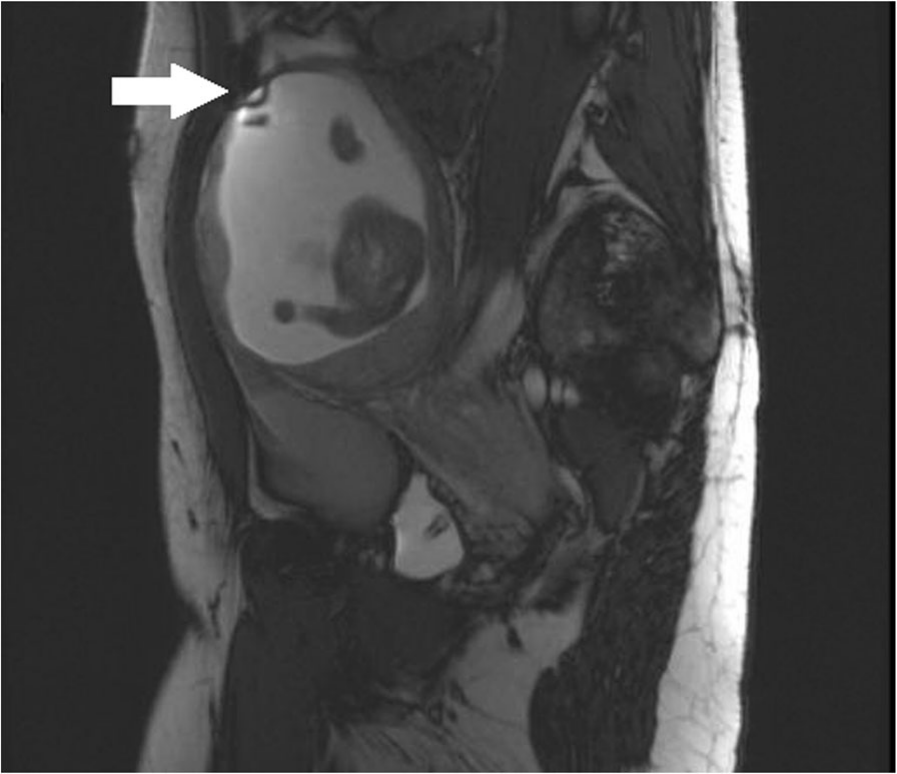
T2-weighted pelvic MRI at 17 weeks gestational age, with thin left uterine horn myometrial thickness (indicated with a white arrow), with a known amniotic band

**Fig. 2:**
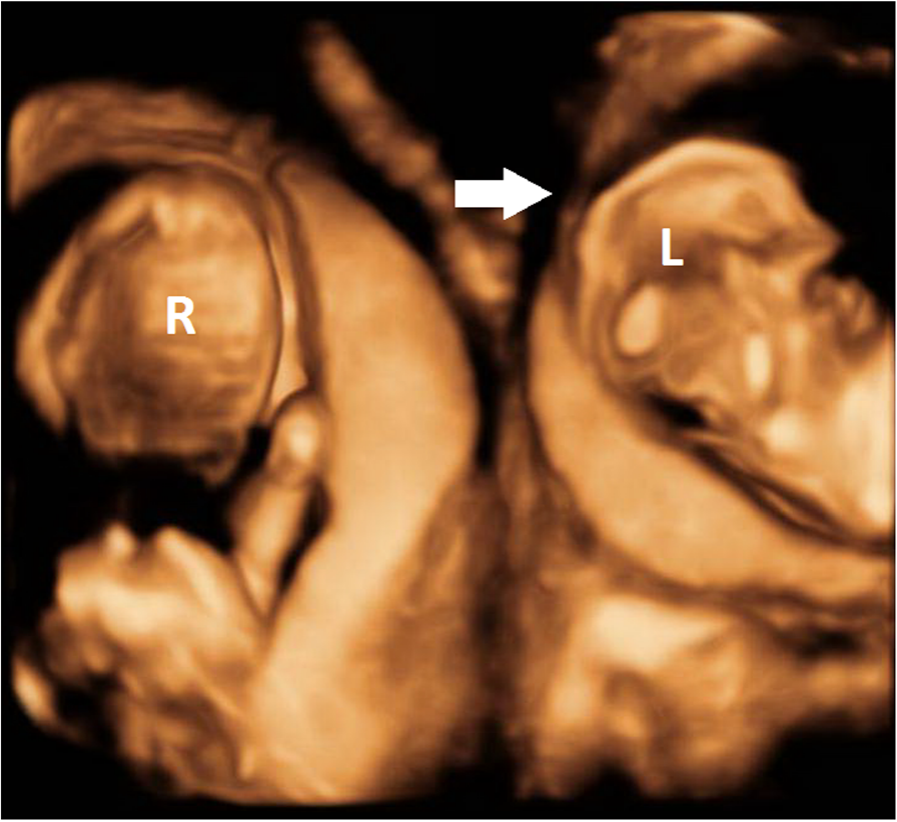
Three-dimensional ultrasound revealing the fetuses in the right (R) and left (L) horns. The thinned myometrial wall is indicated with the white arrow

The patient subsequently presented at 17 weeks gestation with new onset significant pelvic pain. An MRI at an outside hospital demonstrated a thin myometrial wall (thickness not reported) in the left uterine horn without myometrial disruption, but with moderate pelvic free fluid (Fig. 1). The patient’s hematocrit was noted to decline from 32 to 26%, and she was transferred to our tertiary care center for further management. Three-dimensional ultrasound at our institution revealed two separate uterine cavities, each with a live appropriately-grown fetus with normal amniotic fluid. The anatomy of the cervices was difficult to delineate, but a vascular connection was noted between the medial surfaces of each horn. The myometrium over the left horn was noted to be “markedly thinned” superiorly (Fig. 2). Moderate hemoperitoneum was documented, with a 3.6 cm clot adherent to the thinnest portion of the myometrium of the left horn. Concern was raised for rupture of a rudimentary uterine horn. A severe cardiac anomaly was incidentally diagnosed in the fetus in the right unicornuate uterine horn.

**Fig. 3:**
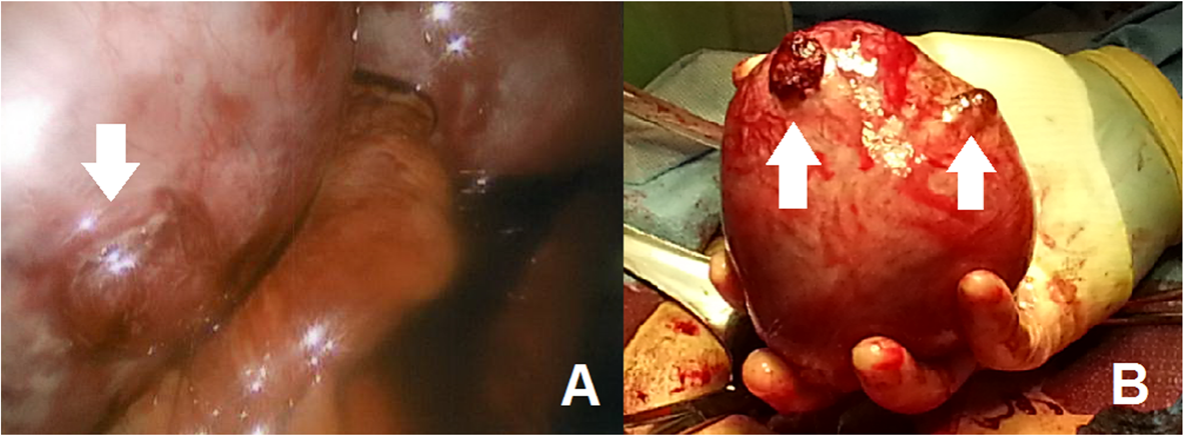
**a** Intraoperative photograph of rupture of the left uterine horn (indicated with an arrow). **b** Photo of excised rudimentary left uterine horn, with two rupture sites indicated with arrows

Given these imaging findings and the patient’s ongoing pain, the decision was made to proceed with diagnostic laparoscopy and left hemi-hysterectomy with fetus in situ. The patient underwent ultrasound-guided selective reduction of the fetus in the left uterine horn using intracardiac potassium chloride. Immediately after this procedure, the patient underwent laparoscopy, at which point she was noted to have a right unicornuate uterus with a rudimentary left uterine horn, with 2 cm rupture on the superior surface with amnion protruding (Fig. 3), and moderate hemoperitoneum. Due to intraoperative bleeding and suspected amniotic rupture during examination of the left horn, the procedure was converted to an abdominal hemi-hysterectomy. During this procedure, a fibrous but narrow band was noted between the uterine horns, which was ligated and transected using bipolar energy. The procedure and the patient’s recovery were uncomplicated.

The pregnancy in the right uterine horn continued postoperatively. At 19 weeks of gestational age, in the setting of a severe fetal cardiac anomaly and high likelihood of preterm delivery complicating surgical correction, the patient underwent laminaria placement and uncomplicated ultrasound-guided dilation and curettage of the right uterine horn.

The patient subsequently spontaneously conceived two singleton pregnancies. Normal anatomical surveys and fetal echocardiograms were noted in each. Both pregnancies were complicated by preterm contractions without cervical change. Eighteen months after her surgery, the patient delivered a healthy, small for gestational age (2,580 g) female infant, followed by a healthy 2,722 g male the following year.

## Discussion and conclusions

Pregnancies implanted in rudimentary uterine horns, though rare, confer significant risk of morbidity and mortality due to uterine rupture and hemorrhage. Heterotopic gestations involving a rudimentary horn (as this patient had) have been reported rarely in the English language literature in the past 20 years, likely due to radiologic advancements allowing for diagnosis prior to pregnancy or gravid rupture [3–5]. None of these reports have included images from MRI and 3-D ultrasound, which are the preferred imaging modalities. These modalities perform similarly for the diagnosis of mullerian anomalies, with sensitivity and specificity of 83–100% [1, 3, 8]. Both modalities are not necessary in every patient with a mullerian anomaly, but given the technical difficulty of diagnosing a rudimentary horn in pregnancy and the obstetrical ramifications, our team chose to collect as much information as possible by performing both in this case.

MRI and 3-D ultrasound are the mainstays of diagnosis of mullerian anomalies, as traditional ultrasound has poor sensitivity (26%) for the diagnosis of a rudimentary horn in both gravid and non-gravid patients [9]. In pregnancy, the distorted uterine anatomy further complicates radiologic assessment of mullerian anomalies. The contour of the uterine fundus, vital to differentiating between a bicornuate uterus from uterine didelphys or unicornuate uterus with rudimentary horn, is obscured when the cavities are distended by advancing pregnancy [3, 9]. Vascular pedicles between the horns, and asymmetrical sizes and/or myometrial thickness of the uterine horns are clues to the diagnosis of a rudimentary horn in pregnancy by MRI or ultrasound, as were identified in this case [3]. Accurate pregestational diagnosis of a rudimentary uterine horn may also require hysterosalpingogram or hysteroscopy (not possible in pregnancy), and/or laparoscopy [3].

This case demonstrates the consequences of an undiagnosed rudimentary uterine horn in pregnancy, and the importance of identifying mullerian anomalies before pregnancy. Many rudimentary horns, however, are undiagnosed prior to pregnancy or may be misdiagnosed, particularly when the myometrium appears symmetrical [1, 3]. When identified prior to pregnancy, rudimentary horns with functional endometrium should be surgically removed to relieve obstruction-related pain when present, and to prevent obstetrical complications [1, 3, 9]. Whether the rudimentary horn communicates with the uterine cavity is irrelevant, as ectopic pregnancies can occur in non-communicating rudimentary horns [3].

In patients with mullerian anomalies diagnosed in pregnancy, particularly those who develop pain, a high index of suspicion should be maintained for a rudimentary uterine horn and possible rupture. Upon diagnosis of pregnancy in a rudimentary horn, immediate surgical management is recommended, though rare late diagnoses and live births (usually preterm) have been reported [2, 3, 9]. Surgeons may use imaging findings—including uterine horn size, evidence of uterine rupture and/or invasive placenta—to plan a surgical approach (laparoscopy versus laparotomy) and prepare for hemorrhage [10]. In patients with the even rarer clinical complication of a heterotopic pregnancy involving a rudimentary horn, surgical management in pregnancy with preservation of the unicornuate uterine gestation is possible, as demonstrated in this case. While the ongoing unicornuate uterine gestations in these cases are subject to the complications associated with this anomaly (pregnancy loss and preterm birth), live births at 36–37 weeks have been described [6, 7].
